# Circulating Th2 cell reduction and Th1/Th2 imbalance are correlated with primary Sjogren’s syndrome-associated interstitial lung disease

**DOI:** 10.1186/s13075-022-02811-z

**Published:** 2022-05-23

**Authors:** Lei Shi, Jia Wang, Hong-Xia Guo, Xiao-Lei Han, Yu-Ping Tang, Guang-Ying Liu

**Affiliations:** 1 Department of Rheumatology, The Second Hospital of Shanxi Medical University, Taiyuan, Shanxi China; 2 Department of Cardiology, The Second Hospital of Shanxi Medical University, Taiyuan, Shanxi China; 3 Department of Mental Health, The Second Hospital of Shanxi Medical University, Taiyuan, Shanxi China

**Keywords:** Primary Sjogren syndrome (pSS), Interstitial lung disease (ILD), Th2 cells, Th1/Th2 ratio

## Abstract

**Objective:**

Primary Sjogren’s syndrome (pSS) is a heterogeneous chronic autoimmune disorder characterized by lymphocyte infiltration of the exocrine glands and the involvement and dysfunction of multiple organs and tissues. Interstitial lung disease (ILD) is the most common type of respiratory system damage. This study ascertained the factors related to ILD in patients with pSS (pSS-ILD), such as altered levels of circulating lymphocyte subtypes.

**Methods:**

Eighty healthy controls and 142 patients diagnosed with pSS were included. The pSS patients were classified into groups with pSS-ILD or pSS without ILD (pSS-non-ILD). Baseline clinical and laboratory data were collected for all subjects, including the levels of lymphocytes measured by modified flow cytometry.

**Results:**

The pSS-ILD patients were older, had higher ESSDAI scores, had higher positivity rates for anti-SSB and anti-Ro52 antibodies, and had more frequent symptoms of respiratory system involvement than pSS-non-ILD patients. pSS-ILD patients had the lowest Th2 cell counts among the three groups. Although the absolute numbers of Treg and NK cells were lower in pSS patients with and without ILD than in the healthy controls, there was no significant difference between the two pSS groups. The Th1/Th2 ratio was significantly higher in patients with ILD than in patients without ILD. Further analysis showed that older age (OR=1.084), lower Th2 count (OR=0.947), higher Th1/Th2 ratio (OR=1.021), and positivity for anti-SSB (OR=3.620) and anti-Ro52 (OR=5.184) antibodies were associated with the occurrence of ILD in patients with pSS.

**Conclusion:**

Decreased circulating Th2 cells and an elevated Th1/Th2 ratio may be the immunological mechanism underlying the development of ILD in pSS patients.

**Supplementary Information:**

The online version contains supplementary material available at 10.1186/s13075-022-02811-z.

## Introduction

Primary Sjogren’s syndrome (pSS) is a chronic progressive autoimmune disease characterized by lymphocyte infiltration of the exocrine glands (mainly the salivary and lacrimal glands), leading to damage to the ductal epithelial and parenchymal structures that results in tissue damage and glandular dysfunction. Although there are common symptoms such as dry mouth and dry eye, pSS is clinically heterogeneous, with the involvement and dysfunction of multiple tissues and organs, such as interstitial lung disease (ILD) [1].

ILD is a chronic inflammatory condition in the lung parenchyma that manifests as different degrees of inflammatory changes and fibrotic destruction of the lung parenchyma; the clinical manifestations, disease progression and prognosis can vary [2]. The reported prevalence of ILD varies substantially (9–75%) according to differences in detection methods and study populations [3–5]. In real-world clinical studies, ILD is also a common manifestation of pulmonary involvement in pSS patients, with an incidence of up to 39.1% [6], and it is closely associated with decreased quality of life and increased mortality in patients with pSS [7]. ILD is also an important factor related to mortality in patients with systemic sclerosis (SSC) (accounting for approximately 35%) [8] and rheumatoid arthritis (RA) [9]. Therefore, it is necessary to further study the risk factors for and pathological mechanism of pSS-associated ILD (pSS-ILD) to find more effective means of achieving an early diagnosis and treatment.

Studies have reported lymphocyte aggregation in lung tissues prior to the appearance of fibrosis in animal bleomycin models of pulmonary fibrosis, suggesting that lymphocytes play an important role in the formation and development of fibrosis [10, 11]. Evidence from many previous studies suggests that T lymphocytes and B lymphocytes account for the majority of the immune cells infiltrating the salivary and lacrimal glands in pSS patients, macrophages and dendritic cells account for the remaining 10% [12], and T lymphocytes play important roles in the pathogenesis of pSS by regulating the immune response [13]. Therefore, disorders of the immune system may be important mechanisms involved in the pathogenesis of ILD in patients with autoimmune diseases, including pSS.

This retrospective study mainly focused on the differences in the levels of peripheral lymphocyte cells between pSS patients with and without ILD to explore the lymphocyte subsets that are closely related to the occurrence and progression of ILD in patients with pSS.

## Materials and methods

### Patients

A cohort of 142 inpatients with pSS who had a confirmed diagnosis of pSS based on the 2002 American–European Consensus Group criteria [14] or 2016 American College of Rheumatology/European League Against Rheumatism classification criteria [15] were recruited from the Second Hospital of Shanxi Medical University between January 2016 and January 2020. High-resolution computed tomography (HRCT) imaging characteristics fulfilled the evidence-based guidelines for the diagnosis and management of ILD published by the ATS/ERS/JRS/ALAT [16] and/or the 2018 Chinese expert-based consensus statement regarding the diagnosis and treatment of interstitial lung disease associated with connective tissue diseases [17]. A contemporary cohort of 80 age- and sex-matched healthy volunteers from the Center of Health Examination in the Second Hospital of Shanxi Medical University were recruited as controls. Subjects who met the following exclusion criteria were excluded from the study: complicated with other connective tissue disease, a history of tuberculosis or severe pulmonary infection and other respiratory diseases, a history of sarcoidosis, malignant tumours, or severe dysfunction of vital organs such as the heart, liver, and kidney.

### Data collection

The baseline demographics and clinical characteristics of all patients were collected from clinical records with a predesigned form. The form contained questions regarding age, sex, duration of pSS, EULAR Sjogren’s syndrome disease activity index (ESSDAI), and clinical manifestations of multisystem involvement, especially respiratory system involvement, such as fever, cough, expectoration, anhelation, stethalgia, haematocyanosis, and Velcro rales.

Laboratory indicators were collected for all patients, including general laboratory items such as the blood count, erythrocyte sedimentation rate (ESR), and C-reactive protein (CRP) level, as well as immunological indicators such as immunoglobulin (Ig), complement (C), and serum-specific antibodies for pSS.

PBMCs isolated from fresh peripheral blood from all participants were stained on the surface and intracellularly with appropriate antibody combinations for the detection of lymphocytes by flow cytometry, including T (CD45^+^CD3^+^CD19−), B (CD45^+^CD3−CD19^+^), CD4^+^T (CD45^+^CD3^+^CD4^+^), CD8^+^T (CD45^+^CD3^+^CD8^+^), and NK (CD45^+^CD3−CD16^+^CD56^+^) cells and CD4^+^T cell subgroups, such as helper T (Th)1 (CD4^+^IFN-γ^+^), Th2 (CD4^+^IL-4^+^), Th17 (CD4^+^IL-17^+^), and regulatory T (Treg, CD4^+^CD25^+^Foxp3^+^) cells [13, 18].

In this study, we used a flow cytometry-based protocol that had a lower chance of generating aberrant results to calculate the absolute numbers of CD4^+^ T subsets based on the following equation: the absolute number of CD4^+^ T subsets = the proportion of each CD4^+^ T subset * the absolute number of total CD4^+^ T cells. Briefly, T cell subset counts (cell/μl) were obtained from fresh blood samples using fluorescent beads in Trucount tubes as internal standards. The total number of CD4^+^ T cells was assessed by flow cytometry (FACSCalibur, Becton Dickinson) according to the stain-and-then lyse-and-wash protocol in the manufacturers’ directions for the BD Trucount^TM^ tubes. Moreover, the cells in 80 μl of heparin-anticoagulated venous blood were stimulated with 10 μl PMA, 10 μl ionomycin (final concentration of 750 ng/ml) and 1 μl GolgiStop before being stained with human anti-CD4-FITC antibodies to facilitate the measurement of the Th1, Th2, and Th17 cells.

### Statistical analysis

Data were analysed with SSPS 25.0 (IBM Software, NY, USA). Numbers and percentages, means ± standard deviations (SDs), or medians (ranges) were used to describe the data collected from all subjects. Differences among groups were analysed using *χ*^2^ tests, independent-sample *T* tests or Mann-Whitney *U* tests. The impact of the potential influencing factors on lung involvement was evaluated with logistic regression. *P* < 0.05 (two-sided) was regarded as statistically significant.

## Results

### Comparison of baseline demographics and clinical characteristics

A total of 142 pSS patients were included and further classified into the pSS-ILD group (*n*=66) and the sex- and duration-matched pSS-non-ILD group (*n*=76). The average age and age at onset of pSS in the pSS-non-ILD group were 55.36±8.51 and 50.03±8.83 years, respectively, and those in the pSS-ILD group were 60.83±8.58 and 55.41±8.41 years, respectively. Patients with pSS-non-ILD were younger than those with pSS-ILD (*P*<0.001). Compared with pSS patients without ILD, patients with pSS-ILD had higher ESSDAI scores [13.5(6.0, 32.0) vs. 3.0(0.0, 10.0), *P*<0.001] and more frequent symptoms of respiratory system involvement, such as cough, expectoration, anhelation, stethalgia, haematocyanosis, and Velcro rales; these differences were statistically significant (*P*<0.05) (Table 1).

**Table 1:** A summary of the baseline demographics and disease characteristics of all enrolled pSS patients

	pSS-non-ILD *n*=76	pSS-ILD *n*=66	*P*
Female, n(percentage)	68 (89.5%)	56 (84.8%)	0.409
Age (years), mean ± SD	55.36±8.51	60.83±8.58	<0.001
Age at onset of pSS (years), mean ± SD	50.03±8.83	55.41±8.41	<0.001
Duration of pSS (months), median(range)	60.00 (0.25, 240.00)	52.00 (2.00, 276.00)	0.492
ESSDAI, median(range)	3.0 (0.0, 10.0)	13.5 (6.0, 32.0)	<0.001
Clinical manifestation, n(percentage)			
Fever	17 (22.4%)	21 (31.8%)	0.205
Cough	19 (25.0%)	44 (66.7%)	<0.001
Expectoration	14 (18.4%)	22 (33.3%)	0.042
Anhelation	11 (14.5%)	24 (36.4%)	0.003
Stethalgia	0 (−)	5 (7.6%)	0.020
Haematocyanosis	0 (−)	7 (10.6%)	0.004
Velcro rale	3 (3.9%)	24 (36.4%)	<0.001
Laboratory indicators, median(range) or n(percentage)			
WBC (×10^9^/L)	5.11 (1.56, 15.19)	5.58 (1.66, 21.78)	0.067
Hb (g/L)	123 (46, 154)	125 (4, 169)	0.571
PLT (×10^9^/L)	215 (11, 511)	203 (39, 759)	0.713
LY (×10^9^/L)	1.63 (0.13, 3.98)	1.55 (0.26, 3.58)	0.286
ESR (mm/h)	27 (1, 120)	37 (2, 120)	0.076
CRP (mg/L)	3.6 (1.0, 138.0)	4.3 (1.0, 114.0)	0.053
IgG (g/L)	14.3 (6.7, 56.1)	15.9 (6.6, 48.2)	0.732
IgA (g/L)	2.7 (0.5, 13.8)	3.1 (0.8, 13.8)	0.197
IgM (g/L)	1.0 (0.3, 11.7)	1.3 (0.3, 22.0)	0.224
C3 (g/L)	0.76 (0.23, 1.48)	0.78 (0.03, 1.49)	0.486
C4 (g/L)	0.18 (0.03, 0.37)	0.19 (0.05, 2.34)	0.496
Anti-ENA	30 (39.5%)	27 (42.2%)	0.745
Anti-Sm	1 (1.3%)	5 (7.8%)	0.059
Anti-SSA (60kD)	31 (40.8%)	35 (54.7%)	0.101
Anti-SSB	5 (6.6%)	13 (20.3%)	0.016
Anti-Ro52	18 (23.7%)	37 (61.7%)	<0.001

*pSS* primary Sjögren’s syndrome, *ILD* interstitial lung disease, *pSS-non-ILD* pSS without ILD, *pSS-ILD* pSS with ILD, *ESSDAI* EULAR Sjögren’s syndrome disease activity index, *WBC* white blood cell, *Hb* haemoglobin, *PLT* platelet, *LY* lymphocyte, *ESR* erythrocyte sedimentation rate, *CRP* c-reactive protein, *Ig* immunoglobulin, *C* complement

The rates of positivity for autoantibodies associated with pSS, such as anti-SSB [13 (20.3%) vs. 5 (6.6%), *P*=0.016] and anti-Ro52 [37 (61.7%) vs. 18 (23.7%), *P*<0.001] but not anti-SSA (60 kD), anti-ENA, or anti-Sm antibodies were significantly higher than those in patients without ILD. There was no significant difference in the blood cell count, ESR, CRP level or remaining immunological indexes, such as Ig, C3, and C4, between the two groups (*P*>0.05) (Table 1).

### Differences in peripheral lymphocyte subsets

The proportions and absolute numbers of the peripheral lymphocyte subsets in pSS patients (including patients with and without ILD) and the age- and sex-matched healthy controls are provided in Table 2. Interestingly, the levels of NK and Treg cells, in terms of both the percentages and the absolute counts, and the proportion of B cells in the peripheral blood of patients with pSS were significantly lower than those in the healthy control group (*P*<0.01).

**Table 2:** Differences in peripheral circulating lymphocyte levels between HCs and pSS patients

	HCs *n*=80	pSS *n*=142	*P*
Sex (female/male), n	64/16	124/18	0.146
Age (years), mean ± SD	55.91±7.17	57.90±8.94	0.090
Numbers of lymphocytes (cells/μl), median(range)			
T cells	1205.25 (646.00, 2415.00)	1175.10 (137.26, 3309.97)	0.340
B cells	177.41 (52.93, 406.00)	213.17 (12.34, 861.13)	0.135
CD4+T cells	655.00 (299.11, 1513.00)	627.74 (28.42, 1902.81)	0.430
CD8+T cells	414.54 (103.00, 1084.48)	445.43 (42.26, 1779.72)	0.925
NK cells	260.50 (52.00, 896.04)	164.00 (13.00, 1107.52)	<0.001
Th1 cells	105.37 (2.69, 515.96)	88.83 (2.14, 1224.46)	0.247
Th2 cells	8.27 (0.96, 27.04)	7.41 (0.28, 71.03)	0.402
Th17 cells	6.44 (0.82, 18.18)	5.24 (0.22, 64.90)	0.264
Treg cells	33.36 (13.26, 70.96)	27.71 (0.14, 139.28)	0.002
Proportions of lymphocytes (%), median (range)			
T cells	72.00 (50.00, 84.72)	71.95 (43.39, 92.87)	0.847
B cells	10.60 (4.65, 23.00)	14.00 (1.76, 46.00)	0.001
CD4+T cells	39.82 (22.86, 57.26)	39.09 (10.23, 59.26)	0.675
CD8+T cells	24.10 (6.00, 48.45)	25.00 (8.73, 62.70)	0.443
NK cells	15.04 (3.00, 31.00)	10.34 (1.00, 39.23)	<0.001
Th1 cells	16.25 (0.25, 49.7)	15.05 (0.32, 83.23)	0.517
Th2 cells	1.22 (0.18, 3.93)	1.08 (0.26, 12.06)	0.773
Th17 cells	0.92 (0.16, 2.78)	0.93 (0.20, 11.02)	0.689
Treg cells	5.13 (2.06, 8.28)	4.26 (1, 18.99)	<0.001
CD4+T/CD8+T	1.64 (0.48, 6.38)	1.48 (0.22, 6.14)	0.335
Th1/Th2	12.93 (0.62, 204)	12.06 (0.15, 121.42)	0.921
Th17/Treg	0.17 (0.03, 0.79)	0.21 (0.02, 2.06)	0.217

*NK* natural killer, *Th* helper T, *Treg* regulatory T, *HCs* healthy controls, *pSS* primary Sjogren’s syndrome

**Fig. 1:**
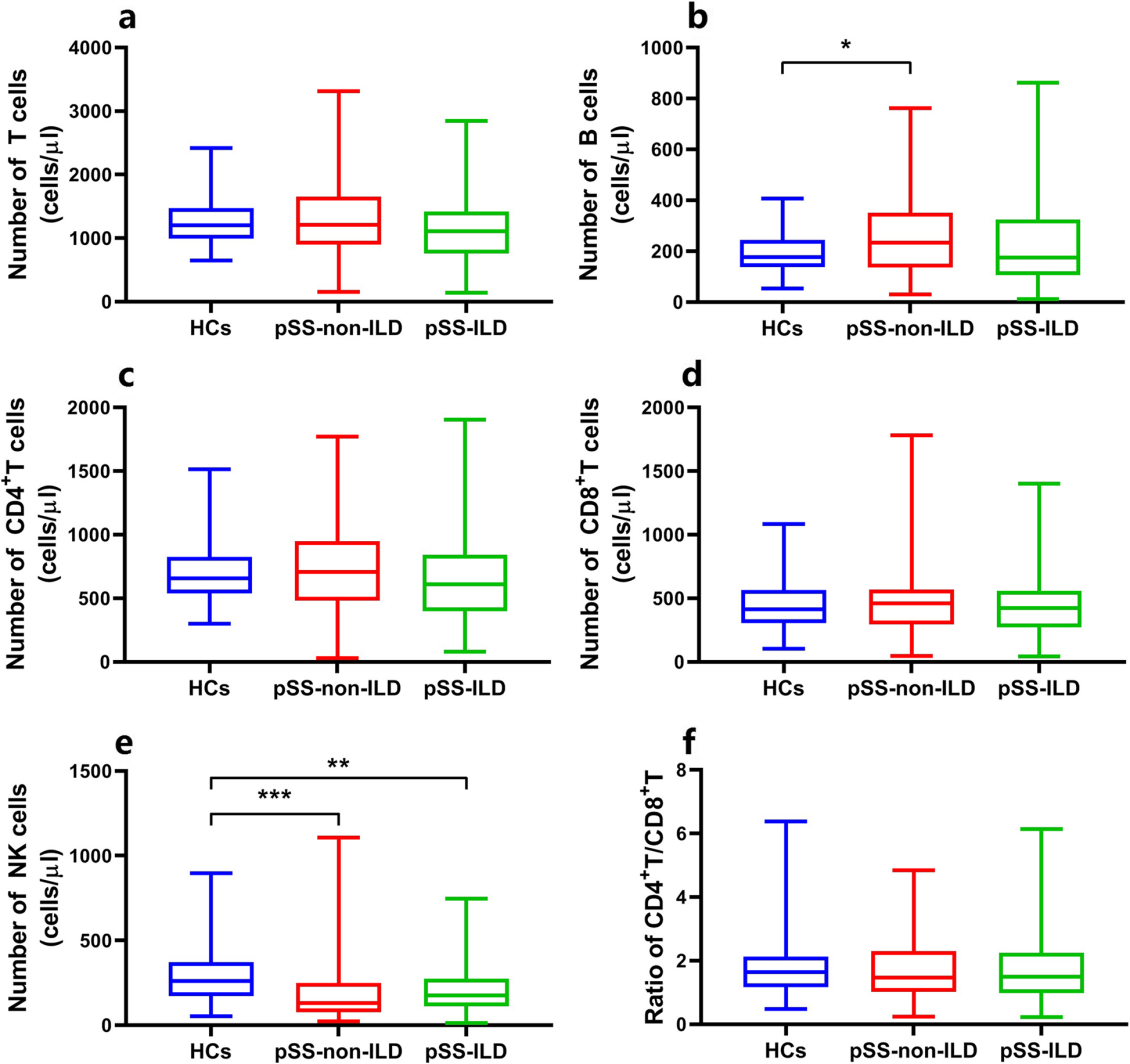
Comparison of the absolute numbers of peripheral lymphocyte subsets among HCs, pSS-non-ILD patients, and pSS-ILD patients. NK, natural killer; HCs, healthy controls; pSS, primary Sjogren’s syndrome; ILD, interstitial lung disease; pSS-non-ILD, pSS without ILD; pSS-ILD, pSS with ILD. *P < 0.05, **P < 0.01, ***P < 0.001. *P* (2-sided tests) *<* 0.05 was considered statistically significant

**Fig. 2:**
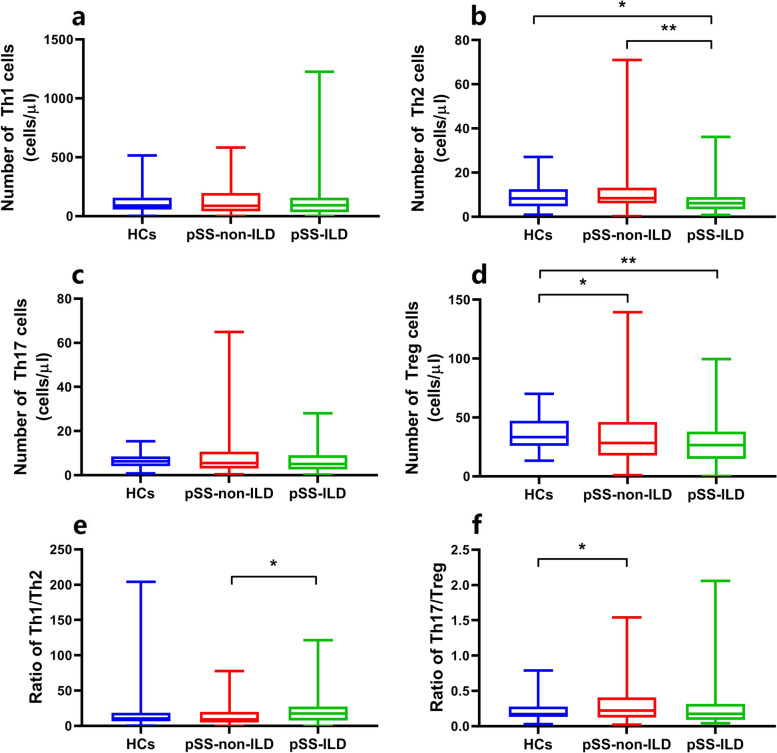
Comparison of the absolute numbers of circulating CD4+ T subsets among HCs, pSS-non-ILD patients, and pSS-ILD patients. Th, helper T; Treg, regulatory T; HCs, healthy controls; pSS, primary Sjogren’s syndrome; ILD, interstitial lung disease; pSS-non-ILD, pSS without ILD; pSS-ILD, pSS with ILD. *P < 0.05, **P < 0.01. Significance values are asymptotic (2-sided tests), and the significance level was *P <* 0.05

The potential role of lymphocytes in the occurrence of ILD in pSS patients was investigated by analysing the clinical data of patients with and without ILD and the healthy controls. The absolute numbers of the lymphocyte subpopulations in the 3 groups were calculated and are shown in Figs. 1 and 2 and Supplementary Figs. 1 and 2. These results verified that both the proportion and absolute number of B cells were significantly higher and that the levels of NK cells were significantly lower in patients with pSS-non-ILD than in the healthy controls, while patients with pSS-ILD had lower absolute counts of NK and Th2 cells than the healthy controls. In addition, compared with patients without ILD, patients with ILD had significantly lower levels (both percentage and absolute count) of Th2 cells and a higher percentage of NK cells. pSS-ILD patients had the lowest Th2 cell counts among the three groups. Interestingly, there was no significant difference in either the percentage or absolute number of Treg cells between pSS patients with and without ILD, although both had lower Treg levels than the healthy controls, which suggested that while lymphocytes may play an important role in the pathogenesis of ILD in pSS patients, Treg cells may not be important targets for the management of patients with pSS [13]. No significant differences were observed in either the percentages or absolute counts of T, CD4^+^T, CD8^+^T, Th1, and Th17 cells among the three groups.

Considering the drastic reduction in Th2 cells in patients with ILD, we next calculated the ratio of Th1 to Th2 cells to determine whether Th2 cells were decreased relative to Th1 cells in patients with pSS-ILD. As shown in Fig. 2e, the Th1/Th2 ratio was significantly higher in patients with ILD than in patients without ILD. The ratios did not differ significantly between patients with or without ILD and healthy controls. The Th17/Treg ratio was also significantly higher in patients without ILD than in the healthy controls, while the difference between pSS-ILD patients and the healthy controls was not statistically significant (Fig. 2f).

### Factors influencing ILD in patients with pSS

To confirm the factors correlated with ILD, this study constructed a logistic regression to further evaluate the correlations between indicators and the differences among the three groups observed in the previous analysis. Each enrolled dependent variable was tested individually in simple regression analyses to obtain the odds ratio (OR). Further analysis showed that the factors correlated with the occurrence of ILD in patients with pSS included older age [OR=1.084, 95% CI= (1.036, 1.133), *P*<0.001], lower Th2 count [OR=0.947, 95% CI= (0.903, 0.994), *P*=0.027], increased Th1/Th2 ratio [OR=1.021, 95% CI= (1.000, 1.042), *P*=0.049], and positivity for anti-SSB [OR=3.620, 95% CI= (1.214, 10.791), *P*=0.021] and anti-Ro52 [OR=5.184, 95% CI= (2.468, 10.886), *P*<0.001] antibodies. There were no significant correlations with the other clinical indicators, indicating that they were not significantly associated with pSS-ILD (Supplementary Table 1). In addition, among the above indicators, only age [OR=1.085, 95% CI= (1.031, 1.141), *P*=0.002] and positivity for anti-Ro52 antibodies [OR=3.927, 95% CI= (1.678, 9.191), *P*=0.002] were significant in multivariate logistic regression.

## Discussion

The management of ILD is challenging due to its heterogeneous nature. Our previous studies have confirmed some of the potential risk factors contributing to pSS-associated ILD, such as cigarette smoking and serum tumour markers [19]. Although different degrees of inflammatory cell infiltration and fibrosis were observed in the alveolar walls and small airways in the lung tissue of 33 patients with pSS-ILD [20], there are still a few questions about the role of circulating lymphocyte cells in the pathogenesis of the disease. We thus initiated a trial aimed at evaluating the role of immune system dysfunction caused by changes in peripheral lymphocyte levels in the pathogenesis of ILD in patients with ILD.

As reported by Wang et al. [21], patients with ILD had higher disease activity scores and more significant symptoms of respiratory involvement, including cough, expectoration, anhelation, and stethalgia. Our analyses clearly demonstrated that the patients with ILD were significantly older than the patients without ILD, which was consistent with the observation made by Zhang et al. [22]. We also found that the rates of positivity for serum anti-SSB and anti-Ro52 antibodies were higher in pSS-ILD patients, and positivity for these antibodies was confirmed to be associated with the onset of ILD in patients with pSS in the logistic regression analysis. It has been reported that the rate of positivity for anti-Ro52 antibodies is high in patients with pSS-ILD, and it has been found to be positively correlated with symptoms such as dry mouth and dry eyes in pSS patients [23–25]. However, neither the saliva nor the serum of patients with inflammatory salivary gland and ductal epithelium lesions in which the Ro52 protein was found to be positive, suggesting that the local rather than the systemic expression of Ro52 was an important cause of inflammation and salivary gland dysfunction [26]. There were no significant differences in the ESR, CRP, Ig, C3, or C4 in patients with and without ILD, which were similar to the findings in the studies conducted by Gao et al. [27] and Wang et al. [21]. However, another study with 87 patients with pSS reported that the levels of the ESR, CRP, IgG, and C3 were significantly higher in patients with ILD than in patients without ILD, while the serum albumin levels were lower [22]. The above controversial results may be related to the differences in the number of included patients and the basic characteristics of patients.

Th2 cytokines, such as transforming growth factor (TGF)-β, interleukin (IL)-4, and IL-13, enhance the development of pulmonary fibrosis by activating fibroblast proliferation and collagen production. GATA-3, a key regulator of Th2 differentiation, regulates the expression of Th2 cytokines by acting as a transcription factor and modifying the chromatin structure of Th2 cytokines [28]. Overexpression of GATA-3 enhances fibrotic processes, perhaps by reducing the level of interferon in the lung tissue, which is consistent with the role of Th2 cells in the promotion of fibrosis [28]. However, this study found that both the absolute count and percentage of peripheral Th2 cells were lower in patients with pSS-ILD than in pSS-non-ILD patients, while the Th1/Th2 ratio was higher. Our results seem to contradict the role of Th2 cells in these processes. In fact, much attention has been given to the increased levels of Th2 cytokines in the lung, which indirectly indicates the increased number of Th2 cells that have infiltrated the lung [28, 29]. Moreover, evidence has shown that the levels of IL-4 and IL-5 and the frequency of Th2 (IL-5) were significantly higher in patients with SSc with lung fibrosis (SScFib+) than in patients with SSc without lung fibrosis (SScFib+), while the ratio of Th1/Th2 in bronchoalveolar lavage (BLA) samples was significantly lower in patients with SScFib+ than in those with SScFib− [30]. Similar results were confirmed in the BLA samples from patients with idiopathic pulmonary fibrosis [31]. Therefore, circulating Th2 cells migrate to lung tissues due to the action of many chemokines and expressed Th2 cytokines, such as TGF-β1 [32], enhancing the processes underlying the development of pulmonary fibrosis, which might explain why the level of peripheral Th2 cells was lower and the Th1/Th2 ratio was higher in patients with pSS-ILD than in pSS-non-ILD patients, while the opposite results were found in the BLA samples from patients with idiopathic pulmonary fibrosis and SScFib+.

Although the absolute numbers of circulating NK cells were lower in pSS patients with ILD than in healthy subjects, there was no significant difference between pSS patients with and without ILD, which means that it remains unknown whether these cells are involved in the pathological process underlying the development of ILD. This is consistent with the findings in a previous study conducted by Izumi et al. [33] NK cells are natural immune effectors that produce a variety of immunomodulatory cytokines in the body and exert cytotoxic effects through cytotoxic granulocytosis. The main components are perforin and serine protease granule B with lymphocyte-specific exocytosis [34], which has been shown to be elevated in lymphocytes infiltrating the lung tissue in patients with idiopathic pulmonary fibrosis and a rodent bleomycin model of pulmonary fibrosis [35]. In patients with diffuse lung parenchymal disease, T lymphocytes may express TGF-β in the lung tissue; TGF-β is an effective anti-inflammatory regulator and profibrotic cytokine [36]. Our previous study found that the serum level of TGF-β1, a key factor in the development of diffuse alveolar injury, alveolar endothelial cell necrosis and pulmonary fibrosis [37, 38], was much higher in patients with ILD than in patients without ILD [32]. Thus, TGF-β-expressing T cells, particularly Th2 and Treg cells, may also be involved in the development of pulmonary fibrosis. The possible explanations for the fewer NK and Treg cells in patients with ILD are as follows: (1) infiltration into the exocrine glands, lung tissues and other involved organs [35, 39]; (2) excessive consumption of peripheral cells caused by disease activity and multiple bacterial and viral infections; and (3) the use of immunosuppressant and hormone drugs, which can directly or indirectly negatively affect those cells [40, 41].

It is worth noting that the results are limited by the fact that this was a single-centre, cross-sectional study with a relatively small number of cases. Further large-cohort longitudinal studies are needed to assess whether changes in Th1 and Th2 levels in the peripheral circulation and pulmonary tissues can be considered predictors of the clinical outcome and progression of pSS-ILD.

## Conclusion

Our data confirmed that older age, positivity for anti-SSB and anti-Ro52 antibodies, and changes in the levels of circulating immune cells, such as NK, Treg, and Th2 cells, are involved in the development of ILD in patients with pSS. In addition, with regard to the altered immune cell levels, fewer Th2 cells and a higher Th1/Th2 ratio are associated with the occurrence of ILD in patients with pSS, which provides a new target for the early diagnosis and treatment of pSS-ILD.

## Supplementary Information

**Additional file 1: Supplementary Table 1.** Single factor logistic regression analysis for pSS patients with ILD. **Supplementary Figure 1.** Comparison of percentages of peripheral lymphocyte subsets among HCs, pSS-non-ILD, and pSS-ILD. NK: natural killer; HCs: healthy controls; pSS; primary Sjogren’s syndrome; ILD: interstitial lung disease; pSS-non-ILD: pSS without ILD; pSS-ILD: pSS with ILD. *P < 0.05, ***P < 0.001. *P* (2-sided tests) *<* 0.05 was considered statistically significant. **Supplementary Figure 2.** Comparison of proportion of circulating CD4+T subgroups among three groups. Th: helper T; Treg: regulatory T; HCs: healthy controls; pSS; primary Sjogren’s syndrome; ILD: interstitial lung disease; pSS-non-ILD: pSS without ILD; pSS-ILD: pSS with ILD. **P < 0.01. The significance level is *P*(2-sided tests) *<* 0.05.

## Data Availability

All data generated or analysed during this study are included in this published article.

## Abbreviations

pSS: Primary Sjogren’s syndrome
ILD: Interstitial lung disease
ESSDAI: EULAR Sjogren’s syndrome disease activity index
Th: Helper T cell
Treg: Regulatory T cell
NK: Natural killer
OR: Odds ratio
SSC: Systemic sclerosis
RA: Rheumatoid arthritis
HRCT: High resolution computed tomography
ATS: American Thoracic Society
ERS: European Respiratory Society
JRS: Japanese Respiratory Society
ALAT: Latin American Thoracic Association
ESR: Erythrocyte sedimentation rate
CRP: C-reactive protein
Ig: Immunoglobulin
C: Complement
PBMC: Peripheral blood mononuclear cell
CD: Cluster of differentiation
IL: Interleukin
IFN: Interferon
Foxp3: Forkhead box protein 3
CI: Confidence interval
TGF: Transforming growth factor
BLA: Bronchoalveolar lavage
SD: Standard deviation
