# Supplementary material for: Circulating Th2 cell reduction and Th1/Th2 imbalance are correlated with primary Sjogren’s syndrome-associated interstitial lung disease
Source: Arthritis Res Ther. 2022 May 23;24:121. doi: 10.1186/s13075-022-02811-z (PMC9125859; doi:10.1186/s13075-022-02811-z)
Supplement: Supplementary file 1 — Additional file 1: Supplementary Table 1. Single factor logistic regression analysis for pSS patients with ILD. Supplementary Figure 1. Comparison of percentages of peripheral lymphocyte subsets among HCs, pSS-non-ILD, and pSS-ILD. NK: natural killer; HCs: healthy controls; pSS; primary Sjogren’s syndrome; ILD: interstitial lung disease; pSS-non-ILD: pSS without ILD; pSS-ILD: pSS with ILD. *P < 0.05, ***P < 0.001. P (2-sided tests) < 0.05 was considered statistically significant. Supplementary Figure 2. Comparison of proportion of circulating CD4+T subgroups among three groups. Th: helper T; Treg: regulatory T; HCs: healthy controls; pSS; primary Sjogren’s syndrome; ILD: interstitial lung disease; pSS-non-ILD: pSS without ILD; pSS-ILD: pSS with ILD. **P < 0.01. The significance level is P(2-sided tests) < 0.05. [file 13075_2022_2811_MOESM1_ESM.docx]

| Supplementary table 1 Single factor logistic regression analysis for pSS patients with ILD | | | | |
| --- | --- | --- | --- | --- |
| Variables | B | OR | 95% CI | *P* value |
| duration (months) | 0 | 1 | [0.994, 1.006] | 0.921 |
| Th1cells (cells/µl) | 0.001 | 1.001 | [0.998, 1.003] | 0.578 |
| Th17 cells (cells/µl) | -0.03 | 0.971 | [0.925, 1.018] | 0.219 |
| Treg cells (cells/µl) | -0.012 | 0.988 | [0.972, 1.005] | 0.157 |
| Th17/Treg | -0.594 | 0.552 | [0.175, 1.743] | 0.311 |
| IgG (g/L) | -0.017 | 0.983 | [0.944, 1.024] | 0.415 |
| IgA (g/L) | 0.057 | 1.058 | [0.904, 1.239] | 0.481 |
| IgM (g/L) | 0.067 | 1.069 | [0.900, 1.270] | 0.446 |
| C3 (g/L) | 0.529 | 1.698 | [0.394, 7.325] | 0.478 |
| C4 (g/L) | 2.047 | 7.747 | [0.288, 208.166] | 0.223 |
| anti-ENA | 0.112 | 1.119 | [0.569, 2.201] | 0.745 |
| anti-Sm | 1.849 | 6.356 | [0.723, 55.888] | 0.095 |
| anti-SSA | 0.561 | 1.752 | [0.895, 3.43] | 0.102 |

pSS: primary Sjögren’s syndrome; ILD: interstitial lung disease; Ig: immunoglobulin; C: complement; Th: helper T; Treg: regulatory T.


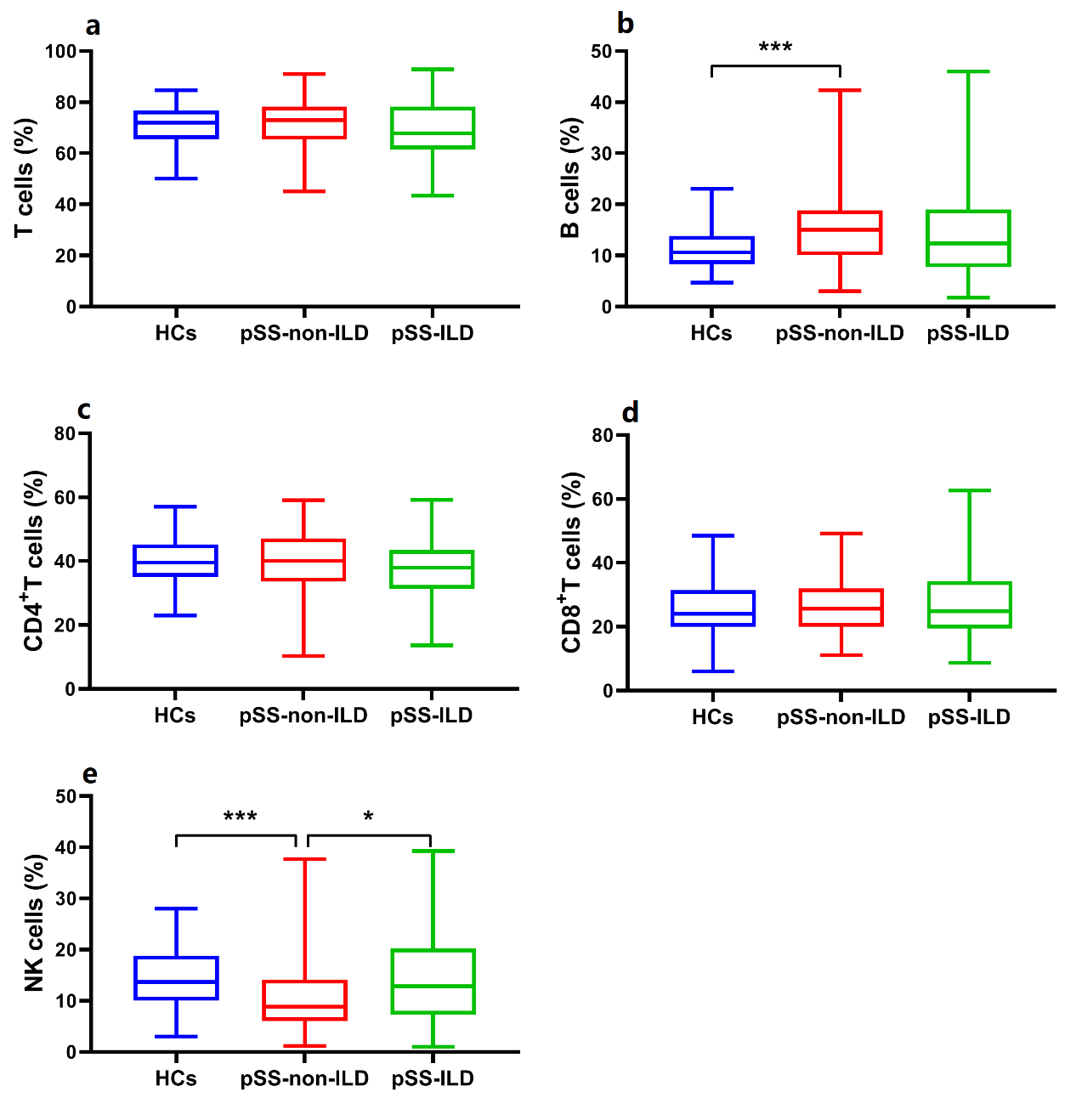


Supplementary figure 1: Comparison of percentages of peripheral lymphocyte subsets among HCs, pSS-non-ILD, and pSS-ILD. NK: natural killer; HCs: healthy controls; pSS; primary Sjogren's syndrome; ILD: interstitial lung disease; pSS-non-ILD: pSS without ILD; pSS-ILD: pSS with ILD. **P* < 0.05, ****P* < 0.001. *P* (2-sided tests) *<* 0.05 was considered statistically significant.


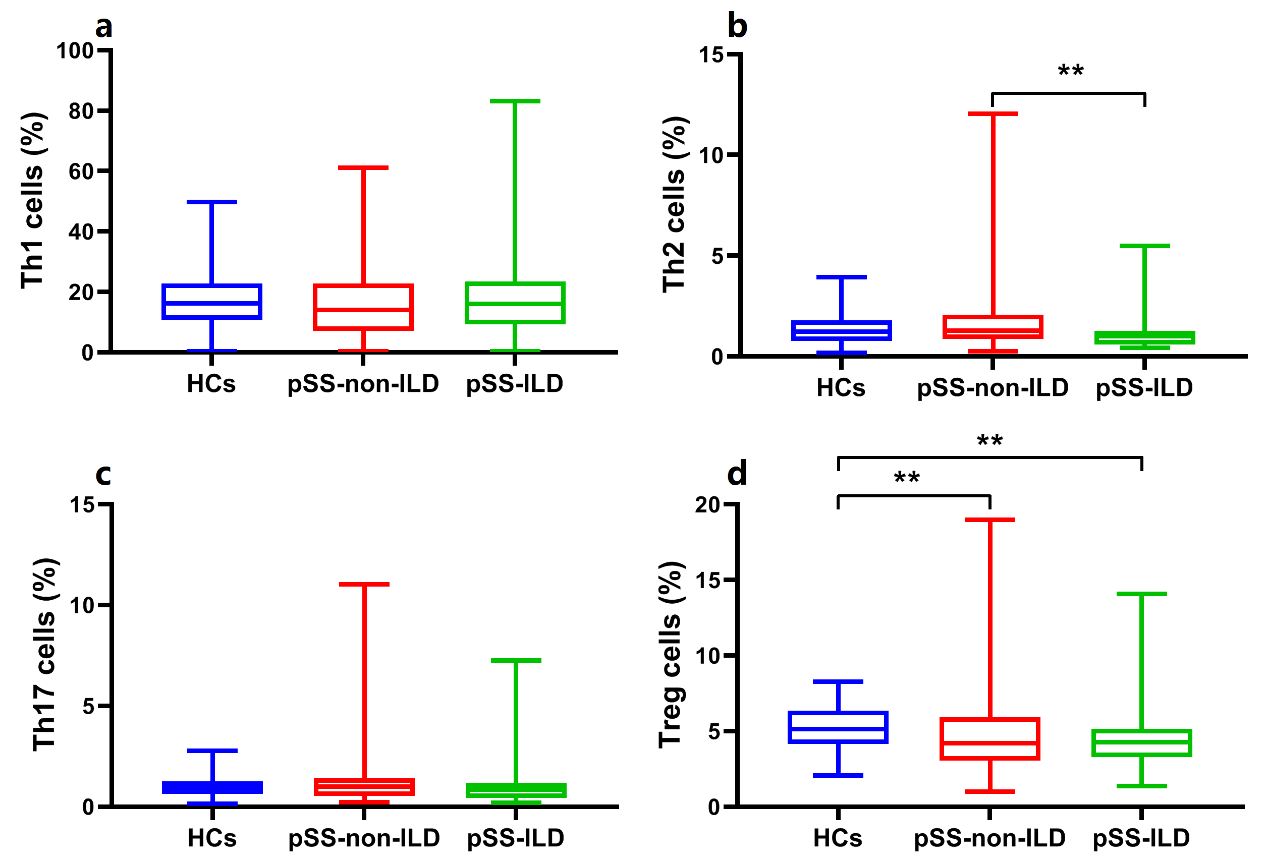


Supplementary figure 2: Comparison of proportion of circulating CD4+T subgroups among three groups. Th: helper T; Treg: regulatory T; HCs: healthy controls; pSS; primary Sjogren's syndrome; ILD: interstitial lung disease; pSS-non-ILD: pSS without ILD; pSS-ILD: pSS with ILD. ***P* < 0.01. The significance level is *P*(2-sided tests) *<* 0.05.
